# BRCA1 p.His1673del is a pathogenic mutation associated with a predominant ovarian cancer phenotype

**DOI:** 10.18632/oncotarget.15151

**Published:** 2017-02-07

**Authors:** Roberta Zuntini, Laura Cortesi, Daniele Calistri, Tommaso Pippucci, Pier Luigi Martelli, Rita Casadio, Elisa Capizzi, Donatella Santini, Sara Miccoli, Veronica Medici, Rita Danesi, Isabella Marchi, Valentina Zampiga, Michelangelo Fiorentino, Simona Ferrari, Daniela Turchetti

**Affiliations:** ^1^ Dipartimento di Scienze Mediche e Chirurgiche, Centro di Ricerca sui Tumori Ereditari, UO Genetica Medica, Università di Bologna, Bologna, Italy; ^2^ Dipartimento di Oncologia ed Ematologia, Azienda Ospedaliero-Universitaria Policlinico di Modena, Modena, Italy; ^3^ Istituto Scientifico Romagnolo per lo Studio e la Cura dei Tumori (IRST) IRCCS, Meldola, Italy; ^4^ Biocomputing Group, BIGEA/Giorgio Prodi Interdepartmental Center for Cancer Research, Università di Bologna, Bologna, Italy; ^5^ UO Anatomia Patologica, Azienda Ospedaliero-Universitaria di Bologna Policlinico S.Orsola-Malpighi, Bologna, Italy

**Keywords:** BRCA1, ovarian cancer, hereditary cancer, VUS, breast cancer

## Abstract

We have investigated the clinical significance of the BRCA1 variant p.His1673del in 14 families from the Emilia-Romagna region of Italy, including 20 breast and 23 ovarian cancer cases; four families displayed site-specific ovarian cancer.

The variant, absent in human variation databases, has been reported three times in BRCA1 specific databases; all probands shared the same rare haplotype at the BRCA1 locus, consistent with a common ancestor.

The multifactorial likelihood method by Goldgar, used to estimate the probability of the variant being causative, gave a ratio of 2,263,474:1 in favor of causality. Moreover, *in silico* modeling suggested that His1673-lacking BRCA1 protein may have a decreased ability to bind BARD1 and other related proteins. All six ovarian carcinomas and two out of four breast carcinomas available showed a loss of the BRCA1 wild-type allele, which in three out of four ovarian carcinomas analyzed by FISH was associated with duplication of the chromosome 17 containing the variant. Although the pathogenicity of the allele is strongly supported by the multifactorial ratio, we cannot exclude that p.His1673del is not itself deleterious, but is linked to another undetected mutation on the same ancestral allele.

## INTRODUCTION

In recent years, the detection of BRCA1 or 2 mutations in patients with breast cancer (BC) or ovarian cancer (OC) has become crucial to tailor their treatment, for instance through more extensive surgery and/or anti-neoplastic drugs that have proven to be specifically effective in BRCA mutation carriers, such as PARP-inhibitors [1]. As a consequence, the request for BRCA1 and 2 genetic testing is rapidly increasing, as is the ability to satisfy these requests thanks to technological improvements, particularly Next Generation Sequencing approaches. Such an increase in both the number and extension of tests (with NGS allowing the analysis also of untranslated regions and deep intronic sequences) will result in an increased detection of Variants of Unknown Significance (VUS). The clinical interpretation of VUS is a challenge for medical geneticists and oncologists, with substantial harm for patients and unaffected carriers deriving from VUS misinterpretation. Furthermore, even when a VUS is correctly interpreted and communicated, risk perception has been shown to be significantly greater than in patients with uninformative results, with a higher rate of prophylactic surgery undertaken [2]. Therefore, the need to classify VUS is particularly urgent, but, unlike other disease genes, functional assays for BRCA1 and 2 are lacking and of limited accuracy. A major advance in the classification of BRCA variants has been the development of a multifactorial likelihood method that combines multiple independent factors to estimate the probability that a BRCA variant is pathogenic [3, 4].

Here, we have used the multifactorial likelihood method to estimate the probability of pathogenicity of a BRCA1 variant detected in multiple breast/ovarian cancer families from our geographical area (Emilia-Romagna, Italy). Furthermore, we have used multiple *in silico* tools to explore the functional effect of the variant, and investigated the mechanisms of BRCA1 loss of heterozygosity (LOH) in ovarian carcinomas occurred in carriers.

## RESULTS

### Genetic analysis and clinical features

**Figure 1:**
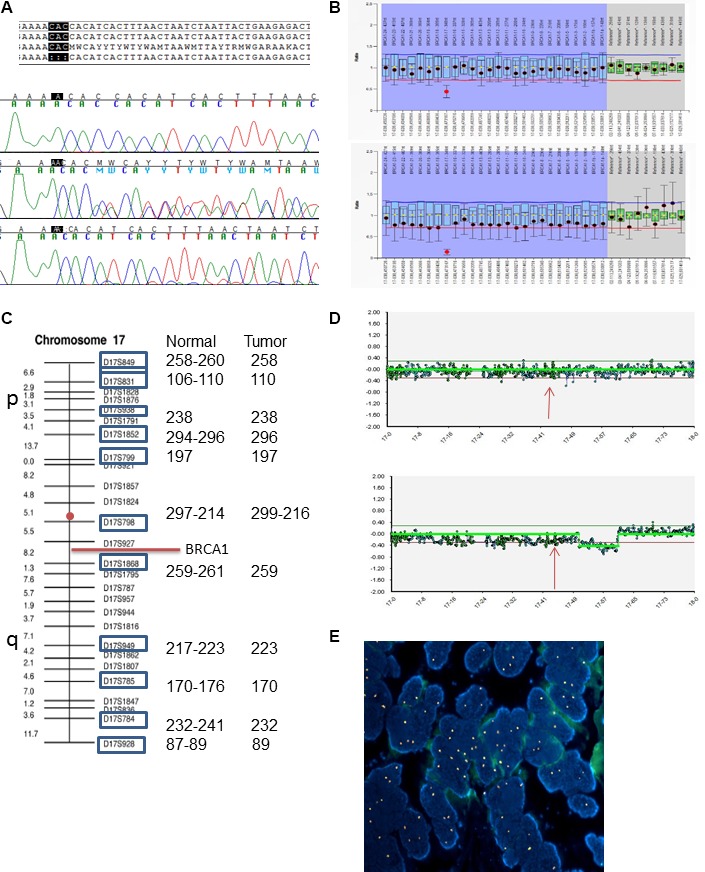
LOH analysis in patient 129-O-14;III-1 (**A**) Sanger sequence alignment with reference sequence; electropherograms shows wild-type sequence (upper), heterozygous sequence (middle) and homozygous deletion (lower). (**B**) MLPA analysis of normal tissue (upper plot) and tumor tissue (lower plot). (**C**) Microsatellite analysis of normal and tumor tissues, showing length of each marker for both tissues. Red circle indicates centromere position. (**D**) CGH array analysis, pattern of chromosome 17 in normal tissue (upper plot) and tumor tissue (lower). Arrows indicate chromosomal position of BRCA. (**E**) Fluorescence *in situ* hybridization (FISH) with centromeric probe for chromosome 17 (Spectrum Orange) showing nuclei with chromosome 17 gains in the ovarian carcinoma of the patient (DAPI, ×100).

In 14 probands undergoing BRCA testing, sequence analysis revealed the tri-nucleotide deletion c.5017_5019delCAC, leading to the deletion of Histidine 1673 in the BRCA1 protein (Figure 1A). The features of families carrying the p.His1673del are summarized in Table 1. As shown, a total of 20 breast carcinomas and of 23 ovarian cancers were reported in these families, with four out of 14 families displaying site-specific ovarian cancer; the ratio of breast to ovarian cancer was 0.87:1.

**Table 1:** Cancer cases in 14 families (bold) and 17 individuals carrying the 1673delHis mutation

Family	Individual	Breast Cancer	Age	LOH	Ovarian Cancer	Age	LOH	FISH Chr 17	Other tumors (age)
**106-O-14**		**0**	**-**	**-**	**2**	**47/78**	**-**	**-**	**None reported**
	III-1	no	-	-	Serous carcinoma G3, IIIc	47	YES	Monosomy	none
**55-O-12**		**2**	**50**	**-**	**2**	**52/53**	**-**	**-**	**NHL (48); *Cervical (60)***
	III-5	no	-	-	Serous carcinoma G3, IIIc	53	YES	Disomy	Diffuse Large B-cell Lymphoma of the tonsil (48)
**129-O-14**		**0**	**-**	**-**	**2**	**45/59**	**-**	**-**	**None reported**
	III-1	no	-	-	Serous carcinoma G3, IIIc	45	YES	Polisomy	none
**91-O-14**		**1**	**52**	**-**	**3**	**46/55/64**	**-**	**-**	**Endometrial cancer (45)**
	III-1	no	-	-	Serous carcinoma G3, IIIc	64	YES	Disomy	none
	III-7	IDC G2, ER+, PGR+, HER2neu-pT1bN0	52	NA	no	-	-	-	none
	III-9	no	-	-	Serous carcinoma G3, Ib	44	NA	NA	none
	II-3	no	-	-	no	-	-	-	Endometrial carcinoma (45)
**298-O-15**		**2**	**51/90**		**1**	**72**			***Colon-rectum (70)***
	III5	no	-	-	Serous carcinoma G3, IIIb	72	YES	NA	
**2915**		**3**	**38/55/57**		**none**	**-**	**-**	**-**	***Brain (66)***
	III-6	IDC G3, ER-, PGR-, HER2neu-pT1bN0	38	YES	no	-	-	-	none
**677**		**0**			**3**	**49/54/69**			**None reported**
	III-5	no	-	-	na	49	NA	NA	none
**726**		**1**	**64**		**3**	**51/52/66**			**None reported**
	III-7	IDC pT2N0 (no grading, nor biomarker assessment)	64		No data available	66	NA	NA	none
**3206**		**0**	**-**	**-**	**2**	**41/41**			**None reported**
	III-1	no	-	-	Serous carcinoma G3, IIc	41	YES	NA	none
**1935**		**3**	**33/37/49**		**none**	**-**	**-**	**-**	**None reported**
	III-7	DCIS	49		no	-	-	-	
**4273**		**1**	**38**		**2**	**48/54**	**-**	**-**	***Lung (55)***
	IV-32	IDC G3, ER+, PGR+ pT1cN0	38		no	-	-	-	
**A250/01**		**1**	**45**		**1**	**40**	**-**	**-**	**None reported**
	III-1	IDC G2, ER+, PGR+, HER2neu-pT1bN0	45	YES	no	-	-	-	
**T112/01**		**3**	**47/54/60**		**none**	**-**	**-**	**-**	**None reported**
	III-4	IDC G3, ER+, PGR+,HER2neu – pT2N3	47	NO	no	-	-	-	
**TR111/01**		**3**	**37/65/65**		**2**	**48/51**	**-**	**-**	**None reported**
	III-2	IDC G3, ER-, PGR-pT3N0	37	NO	Serous carcinoma G3, IIa	47	NA	NA	

All the patients with breast or ovarian cancer who were confirmed to carry the mutation have been listed. Tumors other than breast and ovarian cancer have been reported if they were diagnosed in carriers or first-degree relatives of carriers; those written in italics were reported by the proband but lacked clinical or pathology records to confirm the diagnosis. Abbreviations: IDC: Invasive Ductal Carcinoma; ER: Estrogen Receptor; PGR: Progesterone Receptor. NA: Not Available.

The p.His1673del (rs80358343) variant has been reported once in the BIC database and twice in BRCA Share (formerly UMD-BRCA1 mutations database). In both databases the variant is reported to be of unknown clinical significance; it is not present in the LOVD database, the 1000 genomes database, ExAC nor in EVS. None of the 190 healthy controls from Emilia-Romagna analyzed carried the variant.

In all probands carrying the variant, BRCA1 MLPA analysis apparently showed a heterozygous deletion of exon 16. Actually, the 3′ position of the LPO (left probe oligo) of BRCA1 00780-L00283 maps next to the trinucleotide deletion, causing an apparent deletion of exon 16 as an artifact (Figure 1B).

### Haplotype analysis

All probands shared a common haplotype corresponding to the canonical haplotype designated as number 3 in the paper by Judkins et al. [5] (Supplementary Figure 1). This haplotype is consistently estimated to account for 6% of all BRCA1 alleles in previous studies [5, 6], and in the 1000 Genomes database for the Italian population. Moreover, based on self-reports, all the families, although apparently unrelated, where from a limited area at the boundaries of the Ferrara, Bologna and Modena provinces, suggesting that the deletion arose in a common ancestor living in this area.

### Multifactorial likelihood calculation

**Figure 2:**
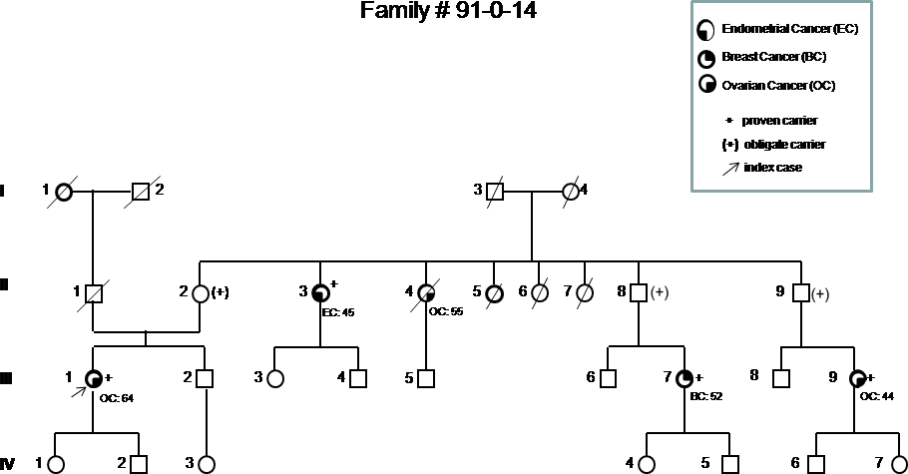
Pedigree of the most representative family carrying the p.His1673del variant, showing the co-segregation of the variant with breast and ovarian cancer in three first-degree cousins (III-1, III-7, III-9) Based on their relationship, the probability that three cousins share a genetic variant by chance would be 1/64. Incomplete penetrance and possible variable expressivity are demonstrated by the presence of the variant in a healthy 87 years old woman (II-2) and in a woman affected by endometrial cancer (II-3).

In order to obtain evidence of the pathogenicity of the variant, we assessed the factors included in the multifactorial model [3]. Among these, co-segregation in the 4 pedigrees with multiple members tested (the largest one is shown in Figure 2) resulted in a combined odds in favor of causality of 16.1:1. As the p.His1673del was never found in patients carrying a pathogenic BRCA1 mutation, co-occurrence analysis gave an odds in favor of causality of 3.57:1. Using the 12-sequence alignment, both the Histidine residues (1672 and 1673) are subject to four amino acid substitutions, with a resulting score of 0.003 [7], regardless of which His is considered. Concerning histopathology, the odds of causation were based on 5 BC cases with available information, and were 0.22:1 for ER receptor status, and 8.75:1 for histology grade. Histopathology data were available for 9 OC cases: the odds of causation for histologic type was 16.47:1, while for histologic grade it was 8.57:1. LOH of the wild-type allele was detected in 2 out of 4 breast carcinomas and in 6 out of 6 ovarian carcinomas analyzed, with odds in favor of causality of 48,312:1.

Overall, the multi-likelihood ratio was 16.1 × 3.57 × 0.22 × 8.75 × 0.003 × 16.47 × 8.57 × 48,312 = 2,263,474:1 in favor of causality, which largely exceeds the cutoff of 1,000:1 adopted to establish that a variant is pathogenic.

### *In silico* modeling

**Figure 3:**
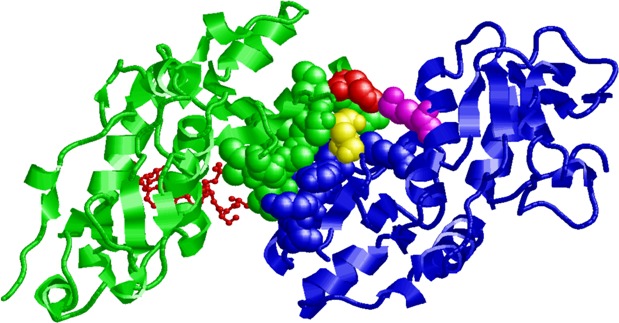
Putative interaction between the BRCT domains of BRCA1 and BARD1 The human BRCT domain of BRCA1 (PDB code: 4Y2G) and of BARD1 (PDB code: 2NTE) are represented in green and blue, respectively. Residues participating in the interaction surface are highlighted with a space-fill representation. Residue His1673 in the BRCT domain of BRCA1 is colored in red and interacts with residues Glu599 (in yellow) and Lys 693 (in magenta) of the BRCT domain of BARD1. The Abraxas phospho-peptide complexed with the BRCA1 BRCT domain is shown in red with a “balls and sticks” representation.

According to software analyses (BDGP Splice Site Prediction, ASSP, Human Splicing Finder and ESEfinder 3.0), the deletion of these three nucleotides does not create any cryptic splice site, nor does it modify potential exonic splicing enhancer sequences. Therefore, we explored the potential effect of the DNA change on protein stability and ability to interact with its natural interactors. We first considered the location of His1673 in the native protein: His1673 is at the C-terminal tip of HELIX 1 (HELIX 1: from residue THR 1658 to 1673, PDB:4Y2G), highly accessible (154 Å^2^, DSSP) and not involved in the phosphoserine binding pocket (about 25 Å apart) (Figure 3). We then used ISPRED3, a state-of-the-art computational tool [8], to estimate the likelihood of each residue in the protein (4Y2G) to be in interaction. The predictor correctly assigns interaction likelihood to residues in the phosphoserine binding pocket; indeed, when the peptide is deleted from the binding pocket, a 64% overall accuracy of prediction is obtained, as depicted by color-codes in Supplementary Figure 2; we therefore assume that the tendency to be at a protein-protein interface computed for other residues is reliable. Among these, His1673 stands out as a putative and important residue in a patch of protein-protein interaction. Among the 10 proteins with known good quality interaction with BRCA1 (STRING 10.0; http://string-db.org), BARD1 is particularly relevant since the heterodimeric complex BRCA1-BARD1 has ubiquitin ligase activity and plays a central role in DNA repair [9]. In the PDB structural database, only the N-terminal heterodimer of BRCA1 and BARD1 (between the RING finger domains) is available (1JM7) [10]. However, considering that the BRCT domains of BARD1 are structurally superimposable with their BRCA1 counterpart, we computed the most stable assembly of the heterodimeric BRCA1-BARD1 protein complex involving BRCT domains. Interestingly, His1673 in the BRCT domain of BRCA1 is predicted to interact (within 5Å distance) with residues Glu599 and Lys693 of the BRCT domain of BARD1 (Figure 3): the suggestion that His1673 of BRCA1 is part of the interaction patch between the two proteins implies that the deleted variant may have a decreased ability to bind BARD1 and other related proteins.

### LOH mechanisms in ovarian cancer

In order to elucidate the mechanisms leading to LOH in ovarian carcinomas, we first performed MLPA analysis on tumor DNA of probands to evaluate the loss of the wild-type allele. Indeed, the absence of the wild-type allele was confirmed through the artifactual homozygous deletion of exon 16, while the other probes showed a reduction of approximately 30%, thus excluding a homogeneous monosomy (Figure 1B). In patient 129-O-14;III-1, CGH-array excluded definite copy number variations of the BRCA1 region, however, a 30% reduction was detected, consistent with MLPA results (Figure 1D). Moreover, microsatellite analysis showed homozygosity of all informative markers when compared with the surrounding normal tissue (Figure 1C). FISH analysis excluded monosomy of chromosome 17. Altogether, these findings suggest that a duplication of the chromosome 17 carrying the p.His1673del occurred, possibly as a compensatory mechanism following the loss of the wild-type chromosome (Figure 1E). FISH in three additional ovarian carcinomas showed monosomy in only one patient, while the other two cases presented chromosome 17 disomy, in spite of the LOH previously detected, supporting the same compensatory mechanisms hypothesized for patient 129-O-14;III-1 (Supplementary Figure 4).

## DISCUSSION

As for more frequent missense variants, the detection of small in-frame deletions is problematic for functional classification and clinical interpretation. Among BRCA1 deletions, the p.Val1688del variant has been classified as pathogenic thanks to evidence gathered by another Italian group [11]. Another single amino acid deletion, located 15 codons upstream, was detected in 14 families from Emilia-Romagna. Like the p.Val1688del variant, this deletion occurs within a tri-nucleotide repeat and, by removing one CAC triplet, leads to the deletion of one of the two Histidine residues at positions 1672 and 1673 (1673 by conventional nomenclature). The multifactorial likelihood method proposed by Goldgar, which has been adopted by the international consortia for VUS classification [3, 4], provided a ratio of 2,263,474:1 in favor of causality. Among the factors considered by the Goldgar model, all but two provided odds in favor of pathogenicity: breast cancer pathology (namely: hormone receptor expression) and conservation. Concerning the former, in fact only two out of 5 breast carcinomas with available information were hormone receptor negative, which is unusual for BRCA1 mutations. However, all 5 breast carcinomas with HER-2 data failed to show amplification, which is in line with that expected for BRCA1 but is not considered in the multi-likelihood model, where it could have compensated the odds calculated for receptors. Regarding conservation, 1672 and 1673 Histidine residues are frequently substituted across species, thus leading to a low score. However, the value of conservation data in assessing the impact of small deletions could be limited, since the removal of one amino acid residue, although functionally non-relevant, could nevertheless alter protein conformation. In addition, it has been demonstrated that some mutations that definitely cause disease in humans are fixed in the genomes of other species, where they are rescued by other *cis* amino acid variants; therefore, a low degree of conservation does not necessarily mean that a change is neutral [12]. The highest odds in favor of causality was provided by LOH analysis; although LOH at the BRCA1 locus has also been described in sporadic ovarian cancer, the LOH rate of 100% detected here represents a strong piece of evidence in favor of causality. Although LOH involved the whole chromosome 17, only one of four ovarian carcinomas analyzed showed monosomy 17: in the other cases, the chromosome carrying the His1673del variant had apparently duplicated after the loss of the wild-type chromosome, leading to disomy (two cases) or polisomy (one case). Furthermore, the CGH-array analysis performed in an ovarian carcinoma showed high genomic instability (Supplementary Figure 3), which further reinforces the hypothesis of a DNA repair defect such as BRCA1 deficiency.

The mechanism of pathogenicity of the p.His1673del variant has been investigated through *in silico* modeling; while no effect was suggested on splicing, the study of protein-protein interactions suggests that His1673 is part of the interaction patch between BRCA1 and BARD1 BRCT domains; therefore, the deleted variant may have a decreased binding capability. Interestingly, the number of ovarian cancer cases in these families exceeded the number of breast cancer cases, and 4 out of 14 families displayed site-specific ovarian cancer, which is in contrast with published genotype-phenotype correlations suggesting a low risk of ovarian cancer associated with mutations falling outside the putative Ovarian Cancer Cluster and/or in the BRCT domain [13].

The fact that the variant is extremely rare world-wide (it is not reported in the major human variation databases) and that all the probands in this study share an uncommon BRCA1 haplotype suggests that it has arisen in a common ancestor living in our geographical area (Northern Italy). Consequently, it cannot be completely excluded that such ancestral allele carries another undefined mutation that is the true causative defect, whereas pHis1673del could be just a marker for the at-risk haplotype.

## MATERIALS AND METHODS

### Family recruitment

A systematic assessment of familial BC risk has been undertaken in the Emilia-Romagna region of Italy since 2012, in order to identify and properly manage women at risk. To this aim, a “Hub and Spoke” regional network was established; the Hubs of this network are third-level Cancer Genetics Centers where genetic counseling is offered and BRCA1/2 genetic testing performed whenever appropriate according to the regional protocol [14].

Fourteen families carrying p.His1673del in BRCA1 were identified in three Hubs of the network: Bologna (5 families), Modena (6) and Meldola (3). Based on self-reports, all the families appeared to originate from the area at the boundaries between the provinces of Ferrara, Bologna and Modena.

### BRCA1 testing

Blood samples were obtained after genetic counseling and informed consent. Genomic DNA was extracted from peripheral blood leukocytes using standard techniques. Complete sequence analysis of BRCA1 and 2 genes was performed by PCR amplification of exons and exon/intron junctions (FastStartTaq DNA Polymerase, Sigma-Aldrich, Saint Louis, Missouri, USA) under standard conditions. PCR products were sequenced on both strands with Big Dye Terminator v1.1 Cycle Sequencing Kit (Thermo Fisher Scientific, Waltham, Massachusetts, USA) and run on automated genetic analyzers. Moreover, analysis of BRCA1deletions/duplications was performed by Multiplex Ligation Probe Amplification (MLPA) using the P002 kit of MRC Holland (Amsterdam, the Netherlands), and data were analyzed using Coffalyser.net software. Mutation nomenclature follows the general recommendations of the Human Genome Variation Society (HGVS): cDNA and protein numbering were based on the reference sequence ID NM_007294.3 and NM_000059.3 respectively.

Targeted p.His1673del analysis in 13 relatives of probands and in 190 healthy controls (blood donors from Emilia-Romagna) was performed by sequencing exon 16 using DNA extracted from peripheral lymphocytes.

### *In silico* analyses

The frequency of the variant was assessed through searches in public databases: 1000 genomes (http://www.1000genomes.org), ExAC (http://exac.broadinstitute.org), BIC (https://research.nhgri.nih.gov/projects/bic/Member/index.shtml), Exome Variant Server-EVS (http://evs.gs.washington.edu/EVS/), UMD (http://www.umd.be/). Databases were last checked on January 18, 2017.

Potential cryptic splice sites and exonic splicing enhancers were investigated by means of BDGP Splice Site Prediction (http://www.fruitfly.org/seq_tools/splice.html), Alternative Splice Site Predictor (ASSP) (http://wangcomputing.com/assp), Human Splicing Finder (http://www.umd.be/HSF3/index.html), and ESEfinder 3.0 (http://rulai.cshl.edu/cgi-bin/tools/ESE3/esefinder.cgi).

The potential effect of the deletion on normal interactions between BRCA1 and its natural interactors was explored using multiple *in silico* tools. First, in order to compute the likelihood of accessible surface exposed residues in the complex depleted of the peptide, we used an in-house tool ISPRED3, available as web server (http://gpcr.biocomp.unibo.it/ispred/) and previously described [15]. We used the recently described BRCA1-BRCT/Abraxas Complex (PDB code: 4Y2G) as a reference structure containing the two interacting BRCT C-terminal domains of BRCA1 [16]. In order to compute the BRCA1-BARD1 structural complex at the level of BRCT domains, we used PDBePISA (http://www.ebi.ac.uk/pdbe/pisa/), an interactive tool for the exploration of macromolecular interfaces. Searching for the most stable assembly of the homodimer of 4YG2, and taking advantage of the structural similarity among 4YG2 and the complex of BRCT BARD1 (PDB code: 2NTE) [9], we obtained the BRCT BRCA1-BRCT BARD1 interacting complex. Solvent surface accessibility was computed with the DSSP program (http://swift.cmbi.ru.nl/gv/dssp/). Structural alignment was computed with JCE (http://www.rcsb.org/pdb/home/home.do). For graphical visualization we adopted RASMOL (http://www.openrasmol.org/).

### Haplotype analysis

Using intragenic polymorphisms covered by standard sequence analysis (c.2196G > A, c.2201C > T, c.2430T > C, c.2731C > T, c.3238G > A, c.3667A > G, c.4427T > C, c.4956A > G, IVS17+66G > A), we defined the haplotypes of patients to assess whether they share a common allele.

### Multifactorial likelihood calculation

The odds of causation of independent factors were estimated as described below, and the product of the odds for each factor was then obtained, according to the method by Goldgar. Cutoffs of 1,000:1 and 100:1 were adopted to classify the variant as deleterious or neutral, respectively [3, 4].

1) Co-Segregation: whenever possible, the search for the mutation was extended to relatives. Co-segregation was analyzed using the Merlin program [17], specifying allele frequency of 0.0001 and penetrance in variant carriers of 0.75, as done by Malacrida et al. [11].

2) Co-Occurrence: Coexistence of the variant with known *BRCA1* pathogenic mutations was assessed in the cohort of 3827 probands from the three centers, of whom 665 carry deleterious *BRCA1* mutations, with an overall frequency of 17.4%. The likelihood ratio was calculated according to the model proposed by Goldgar [3].

3) Conservation: The conservation of p.His1673 and related constrained position LR were assessed according to the multiple-sequence alignments available on the Align GVGD Web site (http://agvgd.iarc.fr/).

4) Histopathology: Pathology records were available for 6 BC and 8 OC, and FFPE specimens for 4 and 6, respectively. For BC histopathologic data, we calculated the odds of causation based on estrogen-receptor status and histologic grade as described by Chenevix-Trench et al. [18], using data from Spurdle et al. [19]. For OC, data regarding grade and histologic type were taken from Lakhani et al. [20].

5) Tumor Loss of Heterozygosity: Loss of heterozygosity (LOH) analysis was performed by PCR amplification of the fragment containing p.His1673del. DNA was extracted from the paraffin-embedded breast and ovarian tumor samples using the QiAmp DNA mini Kit (Qiagen, Hilden, Germany). Polymerase chain reaction (PCR) was performed using primers 16F 5′-ATAACTAGTATTCTGAGCTG-3′ and 16R 5′-ACAACATGAGTAGTCTCTTC-3′. PCR products were sequenced on an ABI-PRISM-3730 genetic analyzer (Thermo Fisher Scientific, Waltham, Massachusetts, USA) using the BigDye Terminator Cycle v1.1 Sequencing Reaction Kit (Thermo Fisher Scientific, Waltham, Massachusetts, USA), according to the manufacturer's protocol. Moreover, analysis of BRCA1 deletions was performed by MLPA. Likelihood ratios were calculated using the probability distribution described by Chenevix-Trench et al. [18].

### Study of LOH mechanisms in ovarian cancer

Complementary approaches were adopted to study rearrangements involving the BRCA1 region in the DNA extracted from fresh-frozen ovarian carcinoma of patient 129-O-14; III-1. Array-CGH was performed using Agilent ISCA 8 × 60 v2 (Agilent Technologies, Santa Clara, California, USA), data were analyzed using BlueFuse Multi v.4.0 software (Bluegnome, Breaks House/Mill Ct, Cambridge CB22 5LD, UK) and heterozygosity was assessed through the analysis of 11 microsatellites mapping to chromosome 17 (panels 23 and 24 by Thermo Fisher Scientific, Waltham, Massachusetts, USA) using automated sequencing, with DNA extracted from surrounding normal tissue as control.

Moreover, chromosome 17 copy number was assessed by FISH of FFPE samples of neoplastic and non-neoplastic tissue from patients 129-O-14;III-1, 55-O-12;III-5, 91-O-14;III-1 and 106-O-14;III-1, using a chromosome 17 probe (CEP 17, Spectrum Orange, Abbott Molecular, USA) as previously described [21]. Tumors with a signal score beyond the cut-off value (set at the mean ± 3SD of non-neoplastic cells) were considered to have gain or loss of chromosome 17.

The study was performed in accordance with the principles embodied in the Declaration of Helsinki; BRCA testing diagnosis and research was approved by the Ethics Boards of the three participating centers.

## SUPPLEMENTARY MATERIALS FIGURES



## References

[1] Parkes EE, Kennedy RD (2016). Clinical application of Poly(ADP-Ribose) polymerase inhibitors in high-grade serous ovarian cancer. Oncologist.

[2] Culver JO, Brinkerhoff CD, Clague J, Yang K, Singh KE, Sand SR, Weitzel JN (2013). Variants of uncertain significance in BRCA testing: evaluation of surgical decisions, risk perception, and cancer distress. Clin Genet.

[3] Goldgar DE, Easton DF, Deffenbaugh AM, Monteiro ANA, Tavtigian SV, Couch FJ, Breast Cancer Information Core (BIC) Steering Committee (2004). Integrated evaluation of DNA sequence variants of unknown clinical significance. Application to BRCA1 and BRCA2. Am J Hum Genet.

[4] Spurdle AB, Healey S, Devereau A, Hogervorst FBL, Monteiro ANA, Nathanson KL, Radice P, Stoppa-Lyonnet D, Tavtigian S, Wappenschmidt B, Couch FJ, Goldgar DE, on behalf of ENIGMA (2012). ENIGMA-Evidence-Based Network for the Interpretation of Germline Mutant Alleles: an international initiative to evaluate risk and clinical significance associated with sequence variation in BRCA1 and BRCA2 genes. Hum Mutat.

[5] Judkins T, Hendrickson BC, Deffenbaugh AM, Eliason K, Leclair B, Norton MJ, Ward BE, Pruss D, Scholl T (2005). Application of embryonic lethal or other obvious phenotypes to characterize the clinical significance of genetic variants found in trans with known deleterious mutations. Cancer Res.

[6] Turkovic L, Gurrin LC, Bahlo M, Dite G, Southey MC, Hopper JL (2010). Comparing the frequency of common genetic variants and haplotypes between carriers and non-carriers of BRCA1 and BRCA2 deleterious mutations in Australian women diagnosed with breast cancer before 40 years of age. BMC Cancer.

[7] Tavtigian SV, Deffenbaugh AM, Yin L, Judkins T, Scholl T, Samollow PB, de Silva D, Zharkikh A, Thomas A (2006). Comprehensive statistical study of 452 BRCA1 missense substitutions with classification of eight recurrent substitutions as neutral. J Med Genet.

[8] Aumentado-Armstrong TT, Istrate B, Murgita RA (2015). Algorithmic approaches to protein-protein interaction site prediction. Algorithms Mol Biol.

[9] Birrane G, Varma AK, Soni A, Ladias JA (2007). Crystal structure of the BARD1 BRCT domains. Biochemistry.

[10] Brzovic PS, Rajagopal P, Hoyt DW, King MC, Klevit RE (2001). Structure of a BRCA1-BARD1 heterodimeric RING-RING complex. Nat Struct Biol.

[11] Malacrida S, Agata S, Callegaro M, Casella C, Barana D, Scaini MC, Manoukian S, Oliani C, Radice P, Barile M, Menin C, D'Andrea E, Montagna M (2008). BRCA1 p.Val1688del is a deleterious mutation that recurs in breast and ovarian cancer families from north Italy. J Clin Oncol.

[12] Jordan DM, Frangakis SG, Golzio A, Cassa CA, Kurtzberg J, Davis EE, Sunyaev SR, Katsanis N, Task Force for Neonatal Genomics (2015). Identification of cis-suppression of human disease mutations by comparative genomics. Nature.

[13] Rebbeck TR, Mitra N, Wan F, Sinilnikova OM, Healey S, McGuffog L, Mazoyer S, Chenevix-Trench G, Easton DF, Antoniou AC, Nathanson KL, CIMBA Consortium (2015). Association of type and location of BRCA1 and BRCA2 mutations with risk of Breast and Ovarian Cancer. JAMA.

[14] Servizio sanità pubblica, Regione Emilia-Romagna: Contributo n. 91/2016: “Protocollo assistenziale nelle donne a rischio ereditario di tumore della mammella e/o ovaio” 2016. http://salute.regione.emilia-romagna.it/documentazione/rapporti/contributi/contributi-n-91-protocollo-assistenziale-nelle-donne-a-rischio-ereditario-di-tumore-della-mammella-e-o-ovaio-2016/view.

[15] Savojardo C, Fariselli P, Piovesan D, Martelli PL, Casadio R (2011). Machine-Learning Methods to Predict Protein Interaction Sites in Folded Proteins. Lecture Notes Comp Sci.

[16] Wu Q, Paul A, Su D, Mehmood S, Foo TK, Ochi T, Bunting EL, Xia B, Robinson CV, Wang B, Blundell TL (2016). Structure of BRCA1-BRCT/Abraxas Complex Reveals Phosphorylation-Dependent BRCT Dimerization at DNA Damage Sites. Mol Cell.

[17] Abecasis GR, Cherny SS, Cookson WO, Cardon LR (2002). Merlin-rapid analysis of dense genetic maps using sparse gene flow trees. Nat Genet.

[18] Chenevix-Trench G, Healey S, Lakhani S, Waring P, Cummings M, Brinkworth R, Deffenbaugh AM, Burbidge LA, Pruss D, Judkins T, Scholl T, Bekessy A, Marsh A (2006). Genetic and histopathologic evaluation of BRCA1 and BRCA2 DNA sequence variants of unknown clinical significance. Cancer Res.

[19] Spurdle AB, Couch FJ, Parson MT, McGuffog L, Barrowdale D, Bolla MK, Wang Q, Healey S, Schmutzler RK, Wappenschmidt B, Rhiem K, Hahnen E, Engel C (2014). Refined histopathological predictors of BRCA1 and BRCA2 mutation status: a large-scale analysis of breast cancer characteristics from the BCAC, CIMBA, and ENIGMA consortia. Breast Cancer Res.

[20] Lakhani SR, Manek S, Penault-Llorca F, Flanagan A, Arnout L, Merrett S, McGuffog L, Steele D, Devilee P, Klijn JGM, Meijers-Heijboer H, Radice P, Pilotti S (2004). Pathology of ovarian cancers in BRCA1 and BRCA2 carriers. Clin Cancer Res.

[21] Giunchi F, Fiorentino M, Vagnoni V, Capizzi E, Bertolo R, Porpiglia F, Vatrano S, Tamberi S, Schiavina R, Papotti M, Bollito E (2016). Renal oncocytosis: a clinicopathological and cytogenetic study of 42 tumours occurring in 11 patients. Pathology.

