# Supplementary material for: BRCA1 p.His1673del is a pathogenic mutation associated with a predominant ovarian cancer phenotype
Source: Oncotarget. 2017 Feb 7;8(14):22640–8. doi: 10.18632/oncotarget.15151 (PMC5410251; doi:10.18632/oncotarget.15151)
Supplement: Supplementary file 1 [file oncotarget-08-22640-s001.pdf]

BRCA1 p.His1673del is a pathogenic mutation associated with a predominant ovarian cancer phenotype

Supplementary Materials

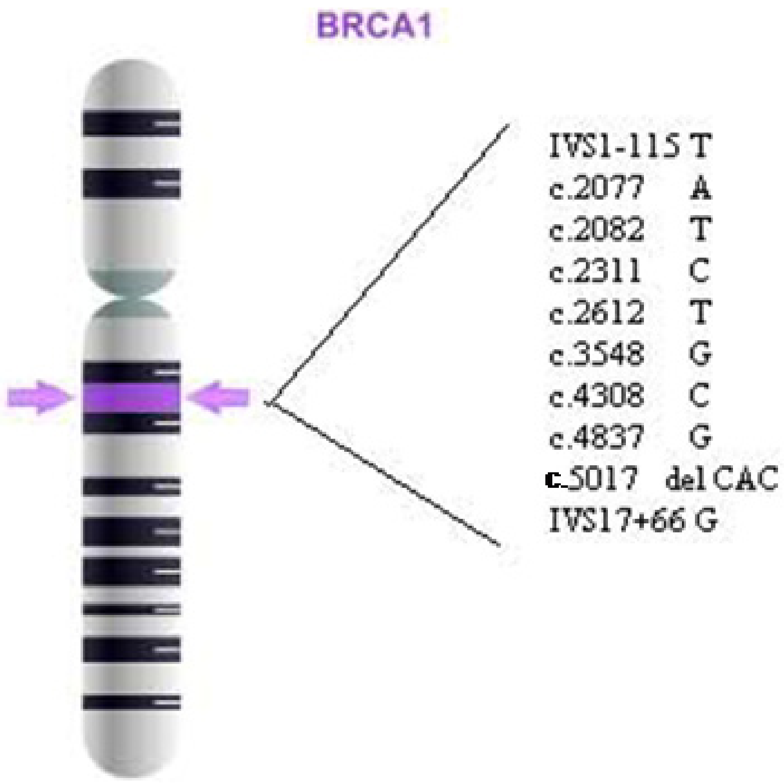

Supplementary Figure 1: Intragenic SNP used to determine common haplotype.

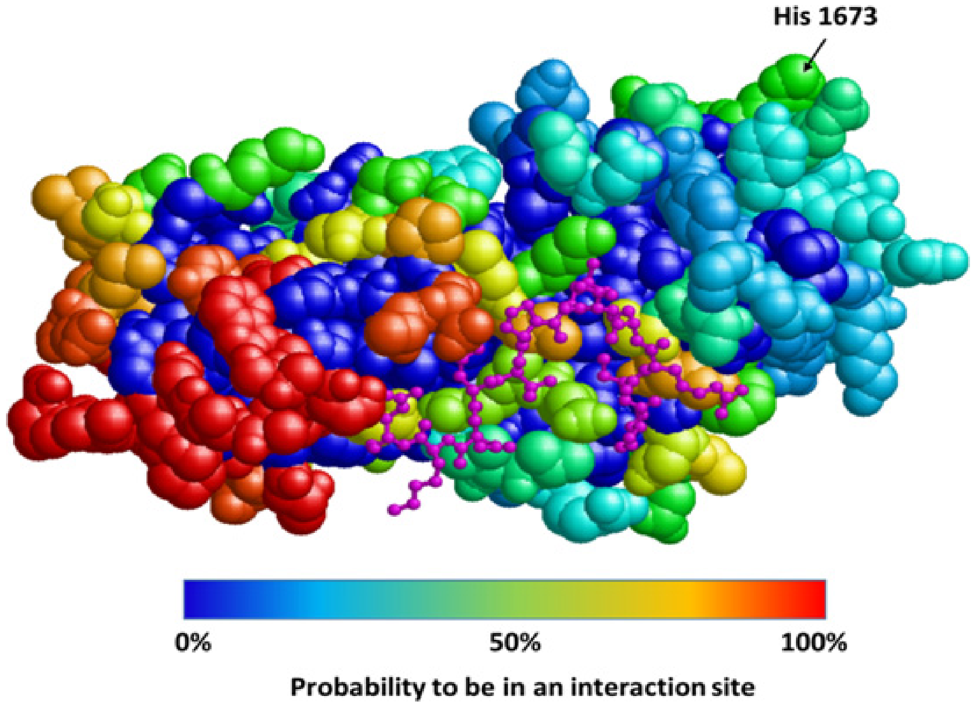

Supplementary Figure 2: Predicted interaction sites in the BRCA1-BRCT domain. Interaction sites were predicted with ISPRED3 (http://gpcr.biocomp.unibo.it/ispred/), using the PDB structure 4Y2G. The "per residue" probability to be an interaction site is colour coded from blue (probability of interaction equal to zero) to red (probability equal to one). The Abraxas phospho-peptide complexed with the BRCA1 BRCT domain is shown in magenta, with a "balls and sticks" representation. The peptide was deleted when predicting its interaction sites, and the efficiency of the tool in predicting the correct interaction was 64%.

Normal tissue

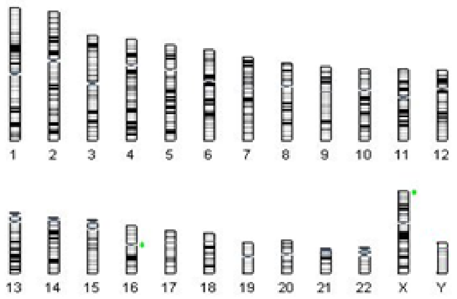

Ch.	Type	Size (bp)
16	Gain	245,250
X	Gain	88,914

Abbreviation: Ch: Chromosome

Tumor tissue

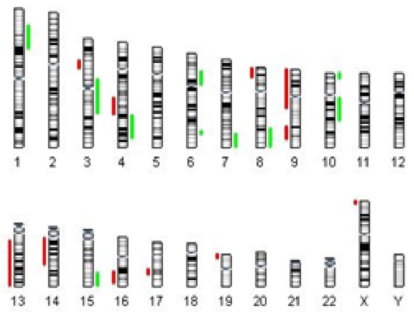

Ch.	Type	Size (bp)
1	Gain	39,670,080
3	Loss	11,929,574
3	Gain	60,319,834
4	Loss	31,057,481
4	Gain	41,576,857
6	Gain	20,993,391
6	Gain	888,299
7	Gain	23,225,038
8	Loss	16,05,592
8	Gain	32,818,954
9	Loss	70,944,868
9	Loss	22,816,081
10	Gain	10,897,376
10	Gain	38,574,873
13	Loss	81,308,550
14	Loss	47,479,756
15	Gain	24,262,224
16	Loss	19,138,689
17	Loss	10,553,46
19	Loss	8,150,778
X	Loss	389,514

Supplementary Figure 3: Karyotype view resulting by CGH analysis of normal and tumor DNA of proband 129-O-14;III-1. In tables are reported all alterations.

Patients		Tumor tissue			Normal tissue		
Family	Individual	% cells 1 copy CEP17	% cells 2 copies CEP17	% cells ≥3 copies CEP17	% cells 1 copy CEP17	% cells 2 copies CEP17	% cells ≥3 copies CEP17
55-O-12	III-5	42,5	45	12,5	50	47,5	2,5
129-O-14	III-1	27,5	32,5	40	35	60	5
106-O-14	III-1	77,5	22,5	0	35	65	0
91-O-14	III-1	52,5	42,5	5	45	55	0

Supplementary Figure 4: Copy number variations of chromosome 17 detected by FISH analysis. Cut-off value to define loss of chromosome 17 (monosomy) was set at > 65% cells with only 1 signal of CEP17, while cut-off value to define gain of chromosome 17 (polisomy) was set at > 15% of cells with 3 or more signals of CEP17.
